# Replacement Adaptor 09106 for patients with a dynamic graciloplasty or patients with sacral neuromodulation and abdominal implantable pulse generators: a retrospective, single centre, Stage 2a/2b development IDEAL case series

**DOI:** 10.1111/codi.16370

**Published:** 2022-10-29

**Authors:** Roman Assmann, Perla Douven, Elbert A. Joosten, Gommert A. van Koeveringe, Stephanie O. Breukink, Jarno Melenhorst

**Affiliations:** ^1^ Department of Surgery Maastricht University Medical Center Maastricht the Netherlands; ^2^ Department of Translational Neuroscience, School for Mental Health and Neuroscience (MHeNS) Maastricht University Maastricht the Netherlands; ^3^ Department of Urology Maastricht University Medical Center Maastricht the Netherlands; ^4^ Department of Anesthesiology and Pain Management Maastricht University Medical Center Maastricht the Netherlands; ^5^ NUTRIM School of Nutrition and Translational Research in Metabolism Maastricht the Netherlands; ^6^ GROW School for Oncology and Developmental Biology Maastricht the Netherlands

**Keywords:** adaptor, dynamic graciloplasty, faecal incontinence, neuromodulation, neurostimulation

## Abstract

**Aim:**

Due to the introduction of a new implantable pulse generator (IPG), the Interstim II, patients with either a dynamic graciloplasty or an abdominally placed IPG for sacral neuromodulation could not undergo surgery to replace their IPG in the case of end of battery life. For these patients, the Medtronic Replacement Adaptor 09106 was created. This retrospective case series aims to study safety and feasibility of the Medtronic Replacement Adaptor 09106 in patients with abdominally placed IPGs.

**Methods:**

Seventeen patients (11 women, six men) received a replacement adaptor with a follow‐up of 6 months. Outcome measures consisted of a bowel habit diary. Adverse events were classified using the Clavien–Dindo classification.

**Results:**

Outcome measures in the bowel habit diaries after replacement (feasibility) did not differ significantly from outcome measures before replacement. Adverse events occurred in four out of 17 patients (24%): two patients initially showed pocket site pain (Clavien–Dindo Grade I), which resolved without intervention. One patient suffered from poor wound closure (Clavien–Dindo Grade II) and one patient had persisting pocket pain (Clavien–Dindo Grade IIIa) for which a pocket revision was performed. Statistical analyses were performed making paired comparisons using a Wilcoxon signed rank test.

**Conclusion:**

The Medtronic Replacement Adaptor 09106 is a valuable option for patients with dynamic graciloplasty or sacral neuromodulation and abdominal IPG and has complication rates similar to replacement of the Interstim without Replacement Adaptor 09106.

## INTRODUCTION

Faecal incontinence is a great burden for which several treatment options have been developed. A few decades ago, two treatment options involving electrical stimulation were introduced: dynamic graciloplasty (DGP) and sacral neuromodulation (SNM).

Graciloplasty was introduced in 1952 to create a neo rectal sphincter and for restoration of anal continence by transplanting the gracilis muscle [1]. However, the results of this technique were mixed due to the presence of a majority of type II muscle fibres in the gracilis muscle [1]. These type II muscle fibres, also known as fast‐twitch muscle fibres, use anaerobic metabolism to create adenosine triphosphate. Anaerobic metabolism is ideal to fuel short bursts of power but is not ideal for enduring exercise like sustaining sphincter tone [2]. In 1988, Baeten and colleagues proposed to stimulate the transplanted gracilis muscle using neurostimulation to induce change from type II to type I (slow‐twitch) muscle fibres [3]. At the same time, Williams et al. developed a similar neurostimulation technique with use of an implantable pulse generator (IPG) to stimulate the gracilis muscle [4]. While the group of Williams chose to indirectly stimulate the obturator nerve by fixing the electrodes directly onto the terminal nerve branches, the group of Baeten fixed the electrodes close to the nerve branches inside the muscle [5, 6]. Both techniques yielded similar results on muscle contraction force, but Williams' technique required more reoperation than Baeten's technique, partly due to leakage of the IPGs [7, 8].

SNM as a treatment modality for faecal incontinence was introduced in 1995 and improved long‐term continence in over 50% of patients [9, 10]. In SNM, an electrode is placed in sacral neural foramen S3 and powered by the aforementioned IPG, also used in DGP [9].

**FIGURE 1:**
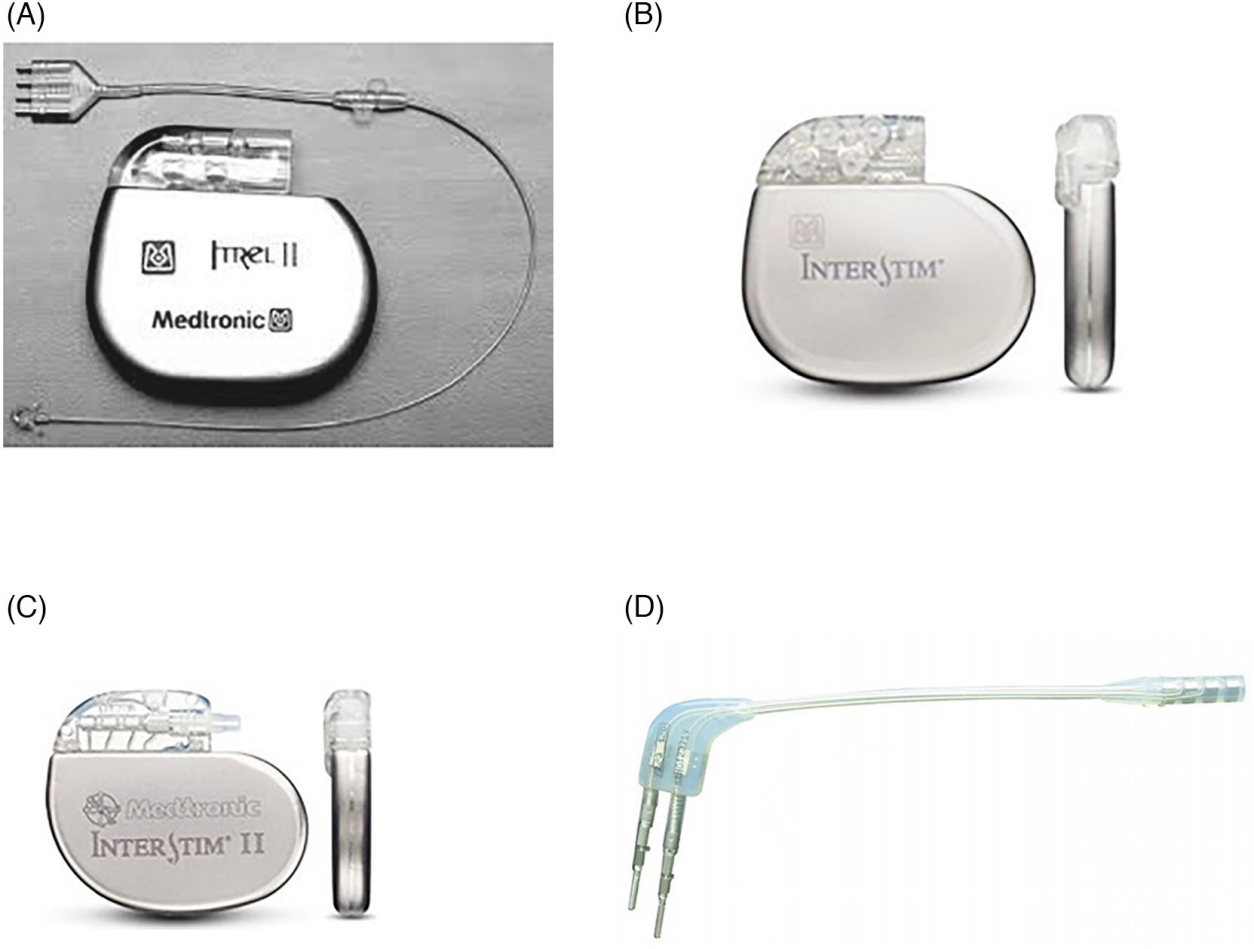
(A) Itrell‐II; (B) Interstim I; (C) Interstim II; (D) Connection piece to connect single SNM lead to two Interstim I access points

Since the first introduction in the early 1990s, several types of IPGs have been released. The first patients were implanted with the Itrel‐I, which only allowed for parameter changes during surgery. In 1994, the Itrel‐II (Figure 1A) was introduced, which allowed the physician to alter parameters using an external programmer. Additionally, this Itrel‐II IPG allowed patients to switch the IPG on and off and to choose between two programmes using a magnet. In 1999, the Interstim I (model 3023) IPG (Figure 1B) was introduced, which empowered patients to switch the IPG on and off using a remote control. This remote control also enabled patients to switch between pre‐programmed parameters and alter amplitude within a certain range [11]. Although IPGs for DGP were always placed abdominally, the placement of the leads for SNM in the sacral foramina allowed for gluteal placement of the IPG, leading to a lower complication rate. In 2006, the Interstim I was replaced by the Interstim II (model 3058) (Figure 1C), which was smaller in size (22 g vs. 42 g, 51 × 44 × 7.7 mm vs. 60 × 55 × 10 mm) [12].

Since 2017, patients with an abdominal IPG for either SNM or DGP to treat faecal incontinence encountered a problem in the case of end of service (EOS), that is, the end of battery life, as the former IPGs (DGP, model 4300/4350 leads; SNM, model 3095 quadripolar extension; Figure 1D) were not produced anymore and the newer Interstim II was not compatible with the leads. One of the main differences between the Interstim I and Interstim II is the reduction in access points from two to one. On the one hand, the DGP consists of two leads placed in the gracilis muscle, which make it perfectly compatible with the Interstim I. On the other hand, SNM uses only one lead, making it compatible with the Interstim II.

To overcome differences between access points and number of leads, a connection piece was needed to connect the single SNM lead to the two Interstim I access points (Figure 1D). In patients with the IPG placed in the buttocks, replacement of the Interstim I by the Interstim II does not pose a problem, since the connection piece can easily be removed to connect the single SNM lead to the single access point of the Interstim II. However, in patients with abdominally placed IPGs, this connecting piece also serves as an extension piece. Therefore, it cannot be removed since the lead would be too short. A previous study by Janssen et al. has demonstrated that lead revision in the case of technical failure has good results on faecal incontinence [13]. Hence, for patients with SNM and an abdominally placed IPG, it would be an option to replace lead, extension and Interstim I by a new lead (gluteally placed) and Interstim II. However, for patients with DGP, this would not be an option because these electrodes are not produced anymore. Moreover, lead removal results in lead breaks in 7.5% of patients [14] and can even lead to life threatening bleeding anterior to the sacral foramen [15].

**FIGURE 2:**
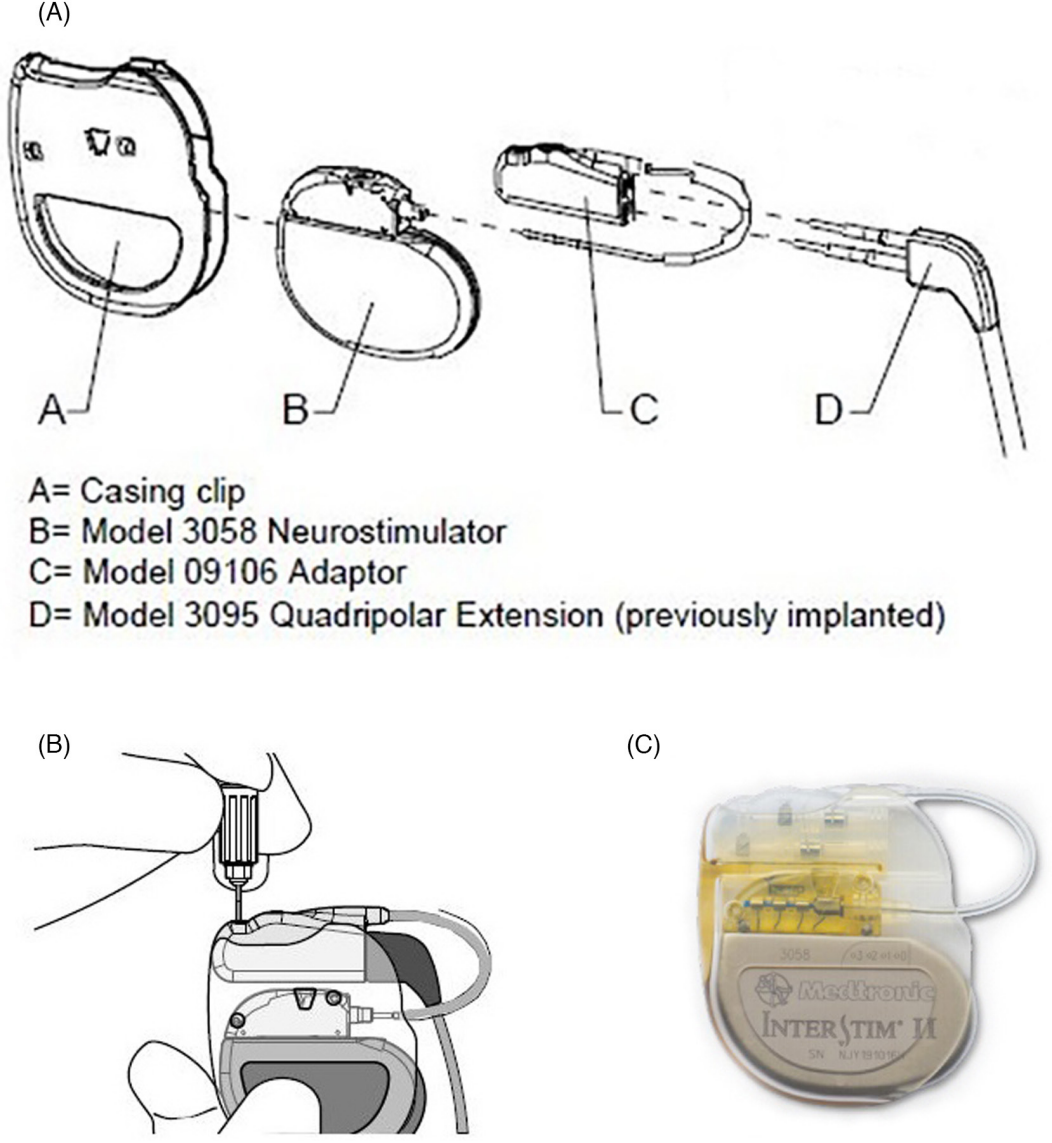
Replacement adaptor 09106

As a solution for patients with DGP and an abdominally placed IPG that reached EOS, and an alternative to performing lead revision for patients with SNM and an abdominally placed IPG, a collaboration between Maastricht University Medical Center+ (MUMC+) and the Medtronic Bakken Research Center created the Medtronic Replacement Adaptor 09106® (RA) (Figure 2). One of the goals when creating this adaptor was to create it with a volume similar to the Interstim I. The RA 09106 received CE marking on 30 January 2018. To date, no previous studies have evaluated the RA 09106.

This study aimed to determine safety and short‐term effects of this new RA 09106 in patients with either a DGP or patients with SNM and abdominal IPGs.

## METHOD

### Study group

This is a retrospective single centre and Stage 2a/2b development IDEAL (Idea, Development, Exploration, Assessment and Long‐term monitoring) case series [16]. All patients with faecal incontinence who received an IPG Interstim II with RA 09106 in MUMC^+^ from February 2018 until April 2020 were included in the study, with a follow‐up of at least 6 months. Ethical approval was granted (METC 2020‐1320) and the research was performed in accordance with the Declaration of Helsinki. Clinical outcome before and after placement of the RA was reviewed retrospectively. Patient characteristics, clinical outcome and adverse events were reported.

### Patient characteristics

For all patients, gender, age, treatment, type of anaesthesia and operating time were recorded (Table 1).

**TABLE 1:** Patient characteristics

Gender	
Female, *n* (%)	11 (64.7%)
Male, *n* (%)	6 (35.3%)
Age, years (mean, range)	64.8 (±10.7; 38.0–81.7)
Treatment	
DGP, *n* (%)	15 (88.2%)
SNM, *n* (%)	2 (11.8%)
Anaesthesia	
General, *n* (%)	16 (94.1%)
Local, *n* (%)	1 (5.9%)
Operating time, min (mean, range)	45.1 (±11.4; 29–79)

Abbreviations: DGP, dynamic graciloplasty; *n*, number; SNM, sacral neuromodulation.

### Clinical outcomes

Patients reported defaecation frequency, faecal incontinence episodes and ability to delay defaecation both before and after replacement by using a bowel habit diary. In our clinical experience, these outcomes are clinically most relevant and most important to patients. Baseline measurements were taken from the last bowel habit dairy a patient filled out before reaching EOS. Follow‐up measurements were taken from bowel habit diaries filled out 6 months after surgery. Patients were followed up in accordance with previous collective experiences [17].

### Adverse events

Adverse events were device‐ and procedure‐related events. All adverse events were scored using the Clavien–Dindo classification [18]. Serious adverse events were scored Clavien–Dindo Grade III or higher and required direct surgical, endoscopic or radiological intervention. Adverse events were scored Clavien–Dindo Grade I or II.

### Procedure for implantation RA

Since patients were already implanted with an IPG (Interstim I), the same pocket was used to place the Interstim II + RA. The abdominal scar was opened under local or general anaesthesia. Then, the pocket, situated behind the anterior wall of the rectus sheath, was opened and the old IPG Interstim I was released from the leads. Subsequently, the leads were connected to the RA and the Interstim II. The combined Interstim II + RA was repositioned in the pocket. The pocket, anterior wall of the rectus sheath and wound were subsequently closed. All surgical procedures were performed by either one of two surgeons. Patients were monitored by contacting them by phone or seeing them physically in the outpatient clinic 1 month, 3 months and 6 months after surgery, after which the regular, yearly check‐ups were resumed.

### Statistical analysis

All statistical analyses were performed using Prism 5 (GraphPad Software). All reported data were continuous and presented as mean ± SD. Data were tested for normality using a Shapiro–Wilk normality test and were found to be non‐normally distributed. Hence, paired comparison was performed using a Wilcoxon signed rank test. A *p* value <0.05 was considered statistically significant.

## RESULTS

### Patient characteristics

Twenty‐two patients underwent successful replacement of the Interstim I by Interstim II + RA between February 2018 and April 2020. Five patients disclosed to their doctor that they had no difference in bowel habit with the Interstim II + RA compared to Interstim I but did not complete a bowel habit diary at 6 months after surgery; these patients were not lost to follow‐up. Therefore, these five patients have been excluded from the analysis. Patient characteristics of 17 patients are shown in Table 1. All patients were treated in day care and were able to leave the hospital on the day of admission. Follow‐up of patients was 6 months.

### Clinical outcome

All patients verbally mentioned that they were satisfied with the replacement by the Interstim II and RA 1 month after surgery. Clinical outcome parameters are shown in Table 2. Defaecation frequency per day did not significantly differ before and after replacement (1.91 ± 1.36 vs. 1.72 ± 1.16; *p* = 0.20). In addition, no significant differences were found in the number of faecal incontinence episodes per week before and after replacement (1.12 ± 1.81 vs. 0.52 ± 1.09; *p* = 0.22). Moreover, the replacement had no significant impact on the ability to delay defaecation before and after replacement (median of 3.5 for both; *p* = 0.50). Finally, there was no difference between impedance before and after surgery (425.6 ± 200.2 vs. 427.4 ± 106.4; *p* = 0.97) and amplitude used to power the electrodes (2.20 ± 0.81 vs. 1.63 ± 0.85; *p* = 0.056).

**TABLE 2:** Efficacy of Interstim II + Replacement Adaptor 09106

	Before replacement	After replacement	*p* value
Defaecation frequency per day (mean ± SD)	1.91 ± 1.36	1.72 ± 1.16	0.20
Faecal incontinence episodes per week (mean ± SD)	1.12 ± 1.81	0.52 ± 1.09	0.22
Impedance of IPG (mean ± SD)	425.6 ± 200.2	427.4 ± 106.4	0.97
Amplitude used for IPG (mean ± SD)	2.20 ± 0.81	1.63 ± 0.85	0.056
Ability to delay defaecation in minutes (median, range)	3.5 (0–720)	3.5 (0–720)	0.50
Adverse events (*n*, %)	NA	4 (23.5%)	NA

Abbreviations: IPG, implantable pulse generator; *n*, number; NA, not applicable.

### Clinical outcome and safety (adverse events)

Adverse events were recorded in four patients. Two patients suffered from pocket site pain initially but this resolved without intervention (Clavien–Dindo Grade I). One patient had poor wound closure, a common complication for this diabetic patient after every procedure. This patient was treated with ciprofloxacin and clindamycin prophylactically and did not suffer from persisting complaints (Clavien–Dindo Grade II). At 3 months’ follow‐up, one patient suffered from pocket site pain, for which she underwent a pocket revision under local anaesthesia (Clavien–Dindo Grade IIIa). After this procedure, the patient was relieved from the pain.

## DISCUSSION AND CONCLUSIONS

This retrospective case series of the RA 09106 demonstrates that the RA is probably safe and that it is a valuable solution for faecal incontinence patients with EOS of the IPG for DGP or SNM. No significant differences were found in bowel habit diary, impedance of IPG and amplitude applied before and after replacement. Twenty‐two patients were eligible for inclusion. However, five of these did not complete a bowel diary. Although we were not able to include these patients in the study because of missing bowel habit diaries, the patients were followed up in the outpatient clinic. They had no problems whatsoever after replacement and mentioned that their SNM/DGP worked just as well as it always had. Complications occurred in four out of 17 patients (23.5%). Two of these patients required intervention (antibiotics and reoperation), which classified the complications as Grade II and IIIa respectively. Two patients were relieved of pocket site pain without intervention (Grade I complication). To date, no literature has been published regarding the RA and therefore we are not able to compare our results to previous studies. However, earlier studies have shown complications in SNM surgery without the RA 09106 in approximately 30% of patients, like the percentage in this study [19, 20].

## LIMITATIONS

There are some limitations to this case series. One of the main limitations is the small number of patients included. This can be explained as the total number of patients eligible for replacement of the Interstim I by the Interstim II plus RA is low. Over the years, more patients will require replacement of the Interstim I with the RA 09106, which means a bigger cohort could be formed. Data of this bigger cohort could confirm the results of this study.

A second limitation of the study is the relatively short follow‐up of 6 months. After these 6 months, patients had yearly check‐ups and were instructed to contact the hospital in case of problems with their IPG, similar to the regular patient contacts. It needs to be emphasized that, despite the limited follow‐up period, none of the patients reported any problems. Follow‐up until the next Interstim replacement would be optimal to study safety and feasibility.

As a third limitation, there is a possibility that Grade I and Grade II complications were missed due to study design. For example, patients may have had pocket site pain for which they took pain medication but may not have mentioned this to their doctor.

## CONCLUSION

In conclusion, this case series demonstrates that the RA 09106 for patients with a DGP or patients with SNM and abdominal IPGs with EOS is a valuable option.

## AUTHOR CONTRIBUTIONS

Roman Assmann: conceptualization; investigation; writing—original draft; methodology; validation; visualization; writing—review and editing; formal analysis. Perla Douven: conceptualization; investigation; validation; visualization; writing—review and editing. Elbert A. Joosten: conceptualization; writing—review and editing; supervision. Gommert A. van Koeveringe: conceptualization; writing—review and editing; supervision. Stephanie O. Breukink: conceptualization; writing—review and editing; supervision. Jarno Melenhorst: conceptualization; writing—review and editing; methodology; supervision; investigation.

## FUNDING INFORMATION

Assmann: unrestricted research grant from Medtronic. Douven: nothing to declare. Joosten: nothing to declare. Van Koeveringe: clinical trial support Medtronic. Breukink: unrestricted research grant from Medtronic. Melenhorst: unrestricted research grant from Medtronic.

## CONFLICTS OF INTEREST

Assmann: unrestricted research grant from Medtronic. Douven: nothing to declare. Joosten: nothing to declare. Van Koeveringe: clinical trial support from Medtronic, consultant and surgical proctor to Boston Scientific, consultancy Solace Therapeutics, clinical trial support Minze Health. Breukink: unrestricted research grant from Medtronic. Melenhorst: unrestricted research grant from Medtronic.

## ETHICAL APPROVAL

Ethical approval was granted (METC 2020‐1320) and the research was performed in accordance with the Declaration of Helsinki.

## Data Availability

The data that support the findings of this study are available from the corresponding author upon reasonable request.
